# The Influence of Atorvastatin Treatment on Homocysteine Metabolism and Oxidative Stress in an Experimental Model of Diabetic Rats

**DOI:** 10.3390/life14111414

**Published:** 2024-11-02

**Authors:** Andreea Clim, Minela Aida Maranduca, Nina Filip, Daniela Maria Tănase, Mariana Floria, Alin Constantin Pinzariu, Irene Paula Popa, Roxana Nemteanu, Tudor Cristian Cozma, Flaviu Ionut Faur, Dragomir Nicolae Serban, Dragoș Viorel Scripcariu, Ionela Lacramioara Serban

**Affiliations:** 1 Department of Morpho-Functional Sciences II, Discipline of Physiology, “Grigore T. Popa” University of Medicine and Pharmacy, 700115 Iași, Romania; clim.andreea@umfiasi.ro (A.C.); minela.maranduca@umfiasi.ro (M.A.M.); alin.pinzariu@umfiasi.ro (A.C.P.); irene-paula_popa@umfiasi.ro (I.P.P.); cozmatudor19@gmail.com (T.C.C.); dragomir.serban@umfiasi.ro (D.N.S.); ionela.serban@umfiasi.ro (I.L.S.); 2 Internal Medicine Clinic, “St Spiridon” County Clinical Emergency Hospital, 700111 Iași, Romania; floria.mariana@umfiasi.ro; 3 Department of Morpho-Functional Sciences II, Discipline of Biochemistry, “Grigore T. Popa” University of Medicine and Pharmacy, 700115 Iași, Romania; 4 Department of Internal Medicine, “Grigore T. Popa” University of Medicine and Pharmacy, 700115 Iași, Romania; 5 Cardiology Clinic, “St. Spiridon” County Clinical Emergency Hospital, 700111 Iași, Romania; 6 Medical I Department, “Grigore T. Popa” University of Medicine and Pharmacy, 700115 Iași, Romania; roxana.maxim@umfiasi.ro; 7 Institute of Gastroenterology and Hepatology, “St. Spiridon” University Hospital, 700111 Iași, Romania; 8 2nd Surgery Clinic, Timisoara Emergency County Hospital, 300723 Timisoara, Romania; flaviu.faur@umft.ro; 9 X Department of General Surgery, “Victor Babes University of Medicine and Pharmacy”, 300041 Timisoara, Romania; 10 Multidisciplinary Doctoral School “Vasile Goldis”, Western University of Arad, 310025 Arad, Romania; 11 Surgery Department, “Grigore T. Popa” University of Medicine and Pharmacy, 16 University Street, 700115 Iași, Romania; 12 1st Surgical Oncology Unit, Regional Institute of Oncology, 2–4 General Henri Mathias Berthelot Street, 700483 Iași, Romania

**Keywords:** lipid profile, homocysteine, oxidative stress, experimental models of diabetes mellitus

## Abstract

Objective: In our experimental study, we evaluated the influence of treatment with atorvastatin on the antioxidant activity of intracellular and extracellular systems factors, homocysteine levels (Hcy), and lipid profiles in obese and diabetic rats. Method: Twenty-one male Wistar rats, aged 6 months, 450–550 g, were allocated into three groups. From the beginning of the study, the first group (G-I, control) received only standard food, while the second and third groups (G II—obese, G III—diabetic) were administered a high-fat diet (HFD) with 2% cholesterol. After 2 weeks of accommodation, the specimens in G-III were injected intraperitoneal (i.p.) streptozotocin (35 mg of body weight, pH 4.5), intervention followed by the onset of type 2 diabetes mellitus. Following confirmation of diabetes onset, the specimens in G III were administered concomitantly with the HFD a daily gavage of atorvastatin 20 mg of body weight/day for 20 days. We measured, at the beginning and the end of the study, the Hcy levels, lipid profile, vitamin B12, B6, folic acid, and various parameters of oxidative stress (OS)—total antioxidant status (TAS), glutathione peroxidase (GPX) and superoxide dismutase (SOD). Results: After treatment with atorvastatin, the lipid profile in G III significantly improved compared to the other two groups, but enzymatic markers of oxidative stress did not closely parallel this trend. However, after the treatment of statin, we observed an important reduction in Hcy values. Conclusion: Our results demonstrate that treatment with atorvastatin can be used not only for its lipid-lowering properties and antioxidant effects but also to reduce Hcy concentration in this experimental model of diabetic rats. Moreover, atorvastatin therapy improves lipid profiles, reduces inflammation, suppresses oxidation, and decreases Hcy levels, potentially preventing major adverse cardiovascular events.

## 1. Introduction

### 1.1. The Importance of Homocysteine in the Wide Picture of Cardiovascular Diseases

The Framingham Heart Study still serves as a very powerful tool for assessing the risk for cardiovascular disease [1]. “Classical” risk factors involved in the physiopathology of CVDs—both modifiable and non-modifiable—include age, sex, stress, obesity, dyslipidemia, blood pressure, smoking, alcohol, diabetes mellitus, and physical inactivity. Furthermore, non-traditional factors such as atherosclerosis, oxidative stress (OS), homocysteine (Hcy) concentration, and inflammation are cited in the development of CVDs [2,3,4].

Hcy is a non-proteinogenic α-amino acid, the result of methionine demethylation in muscles, liver, and other tissues. The normal serum Hcy concentrations range from 5 to 15 µmol/L. Recent research defined precise cut-off values, classifying Hcy levels as mildly elevated (15–30 µmol/L), moderately elevated (31–100 µmol/L), and severely elevated (more than 100 µmol/L) [5]. A moderate increase in Hcy plasma levels is considered an independent predictive factor for several cardiovascular-related statistics: early disease onset, morbidity, and mortality [6,7,8].

Transmural impairment of the vascular wall is the foundation for atherosclerosis. Epidemiological studies demonstrated a positive correlation between Hcy levels and early stages of atherosclerosis: directly, by measure of carotid vascular wall thickness (CIMT), and indirectly, by correlating disease prevalence to Hcy concentration independently of other cardiovascular risk factors [9,10,11,12,13,14]. One mechanism through which hyperhomocysteinemia (HHcy) interferes with endothelial health is a reduction in the availability of nitric oxide (NO), both in directly—activating protein kinase C—and indirectly—accumulation of asymmetric dimethylarginine, which inhibits the local NO synthase [15,16]. A further damaging effect of Hcy on endothelium is mediated by an increase in oxidative stress [17]. Alteration of the lipid profile is constant in atherosclerosis; Hcy negatively influences the retrograde transport of cholesterol, leading to lower high-density lipoprotein cholesterol levels [18]. Favored by a pro-oxidative milieu under elevated plasma Hcy, oxidized particles of low-density lipoprotein cholesterol (ox-LDL) initiate the first stage of atherosclerotic plaque [19].

Hcy level could represent, on the one hand, a biomarker for the extent of cardiovascular damage or, on the other hand, an independent factor in the progression of CVDs [20]. Concerning the significance of homocysteine as a cardiovascular risk factor, there has been a lot of controversy in the past, yet some still persist in the present [21,22]. Not only the role of Hcy as a determinant of the disease is challenged by some authors, but the same can be said about the traditional route of lowering this biomarker—by balancing the B-vitamin status [23]. In light of this ever-existing lack of agreement, our team came up with a model of cardiovascular double-challenged rodent (by induction of obesity and diabetes mellitus type 2) together with the aim of assessing the efficacy of statins (an almost dangerously widespread medication class) on amelioration of this complex metabolic syndrome.

### 1.2. Diabetes Mellitus Type 2 and Obesity Challenge the Homeostasis of the Endothelium

Characterized by hyperglycemia, dyslipidemia, insulin resistance, and hyperinsulinemia, TSDM represents a major risk factor for cardiovascular mortality and reduced global life expectancy [24]. In diabetic patients, vascular endothelial cells are typically damaged by OS, resulting in leaky endothelial junctions, intensification of vascular wall permeability, and ultimately promoting the progression of vascular disorders [25].

Obesity induces persistent inflammation, increasing the risk of atherosclerosis. This pathological mechanism involves the stimulation of adipokines, cytokines, and increased aldosterone serum levels. Adipokines such as leptin, IL-6, resistin, and monocyte chemoattractant proteins stimulate and induce the migration of monocytes and macrophages to adipose tissue, promoting inflammation in visceral and, therefore, systemic adipose cells. This contributes to OS, disturbs lipidic and glycemic metabolism (induces insulin resistance), endothelial dysfunction (ED), and consequently, a hypercoagulability status; all of these factors play an important role in atherosclerosis [26].

Vascular endothelium and vascular health play the principal role in CVDs. It is considered an active monolayer that covers the cells of the vascular lumen, separating the vascular wall from the circulating blood. Early disruption of the mechanisms involved in vascular homeostasis equilibrium determines endothelial dysfunction (ED) [27]. The important role of inflammation in the development of atherosclerosis has been discussed for more than 20 years [28].

### 1.3. Atorvastatin—A Thoroughly Understood Drug. But There Is More to Unveil

Recent data have shown the crucial role of treatment with inhibitors of 3-hydroxyl-3-methylglutaryl coenzyme A-reductase (statins) in reducing CVD risk. These play a central role, not only through a pleiotropic effect, but they also variably reduce inflammation independently from lipid metabolism interference, confirming the role of inflammation in atherosclerosis. Atorvastatin attenuates macrophage foam cell formation in the context of controlling scavenger receptors, oxidized LDL uptake, and cholesterol efflux [29,30]. Atorvastatin is a potent antioxidant and has anti-inflammatory properties, autonomous of its lipid-lowering characteristics. It is usually employed in medical practice, in cardiovascular therapy, has hypolipidemic effects, is easily tolerated by patients, and adverse events are rare.

Aside from shifting the local cytokine balance, statins can reduce endothelial damage by increasing the viability of endothelial progenitor cells and inhibiting endothelial cell apoptosis [31,32]. Therefore, atorvastatin can potentially slow the advancement of atherosclerotic plaques and reduce their vulnerability to rupture by influencing the activation of endoplasmic reticulum stress. Additionally, atorvastatin acts as a hypocholesterolemic agent, offers hepatoprotective effects, and improves cardiovascular function by modulating OS, nitric oxide (NO) levels, and Hcy levels in rat models [33,34]. Many epidemiological studies proved that the risk of developing cardiovascular disease increases significantly with increasing plasmatic Hcy at any level above the normal range.

Despite a beneficial effect arising from minimal therapeutic doses of atorvastatin or simvastatin were proposed on Hcy values in multiple clinical studies, the results remain controversial [35,36,37,38,39]. Furthermore, the 5-year predictive risk for cardiovascular adverse events in patients treated with statin is directly impacted by HHcy severity, regardless of regime intensity [40]. The predictive value of homocysteine regarding cardiovascular adverse events remains significant after adjustment for the presence of diabetes mellitus [41].

These are arguments towards the still-existing need for understanding the influence of statin therapy on the concentration of homocysteine, the main outcome of interest in our experiment design.

### 1.4. Aim of Our Study

In light of the current state of the literature, our team chose a well-proven diabetic and obese model of rodents in order to assess the effect of a statin still used in clinical practice. This model raised the most interest to our team since the cardiovascular burden comes from two directions: one is diet-determined obesity, and the other is the damage mediated by altered metabolism and advanced glycation products in the context of impaired glycemic control.

The aim of this study was to determine the impact of atorvastatin in obese rats challenged with artificial-induced diabetes mellitus in comparison to just obese rats. Consistent with the most often indication atorvastatin is prescribed for, the main outcome of interest was represented by traditional (lipid profile) and non-traditional (homocysteine, oxidative stress) cardiovascular risk factors. The secondary outcome was the B vitamin complex in light of altered homocysteine metabolism.

Therefore, in this experimental research, we examined the potential effect of atorvastatin on the Hcy plasma levels, OS, and lipidic profile in obese and diabetic rats. To determine the OS and the response of the intracellular antioxidant protection mechanism in diabetic models of rats, we evaluated the activities of superoxide dismutase (SOD) and glutathione peroxidase (GPx) levels in red blood cells. Conversely, for the extracellular arm, we measured the total antioxidant status (TAS) plasma levels. To replicate a human predisposition to fat, high-fat diets and rodents (HFD) have been employed to mimic obesity in humans; it is thought that these animal models of obesity display a closer physiology to humans, better than the genetically modified ones do. When it comes to HFD-induced body weight gain, Wistar shows a relatively narrow distribution, qualifying it as the best candidate for our experiment [42].

## 2. Materials and Methods

### 2.1. Animals

Twenty-one (21) Wistar rats, aged 6 months (450–550 g), male, were procured from the Cantacuzino Institute, Bucharest, Romania, as a part of more complex experimental studies. The animals were housed in CEMEX at “Grigore T. Popa” University of Medicine and Pharmacy, Iași, Romania, facility conditioned at 20 ± 4 °C, 50 ± 5% humidity, on a 12 h light/12 h dark cycle). Also, the animals had individually ventilated cages and access ad libitum to water and food. After 14 days of acclimatization, the animals were included in the experimental protocols.

### 2.2. Ethical Approach

The experimental investigation was according to the European Directive 2010/63/EU. Therefore, it is approved by the ethics committee of the university (No. 277). Also, we have authorization from the Romanian National Sanitary Veterinary and Food Safety Authority (No. 61).

### 2.3. Experimental Design and Animal Treatment Groups

**Figure 1:**
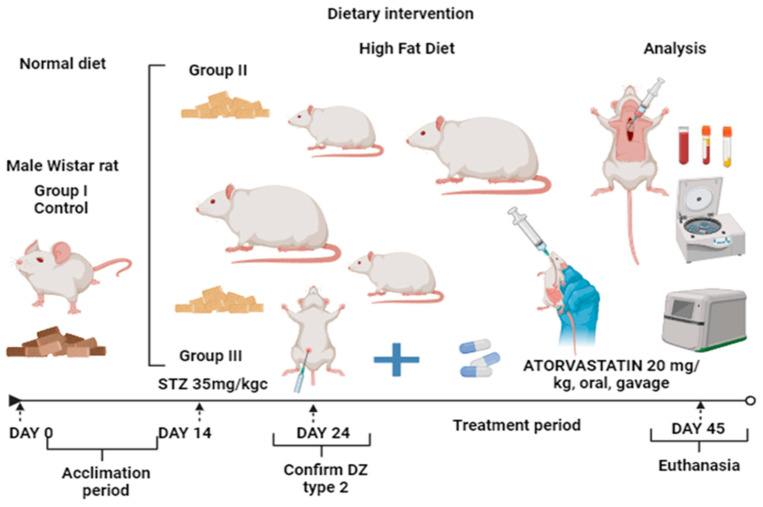
Experimental design. In this experimental study, we included twenty-one Wistar rats. After 14 days of acclimatization, the rats were allocated into 3 groups: group I (control group reserved standard diet), group II (with obesity, the animals constantly received special food, with high-fat diet with cholesterol 2%), and group III (initially with obesity, and then diabetes mellitus, the animals constantly received special food, with high-fat diet with cholesterol 2%). In group III, after 14 days, we administered treatment with STZ to induce diabetes mellitus (35 mg/kg of body weight). We evaluated the levels of glycemia at 7 and 10 days following the injection and confirmed the diagnostics of T2DM at 10 days. After the confirmation, we administrated atorvastatin treatment in dosages of 20 mg/kg of body weight, orally, by gavages, every day for the next 20 days. In the final experiment, we sampled blood by cardiac puncture. The blood was stored in special vacutainer tubes with a clot activator solution for biochemical assays.

To evaluate biochemical changes in the administration of atorvastatin on OS, the rats were grouped into three groups of equal numbers (*n* = 7, ages 6 months, weight 450–550 g for groups II and III with obesity and 150–200 g for group control). After 14 days of acclimatization, all the rats received treatment according to the experimental protocols, and they had access to water without restriction. The animal experimental short design is shown in Table 1 and Figure 1.

Atorvastatin (Sortis, Pfizer, No NM4027) was mixed with distilled water and administered once a day orally, by gavages, using a sterile dispositive for every rat (16 G × 1.1/2, Popper and Sons). Prior to initiating the treatment with atorvastatin, we diagnosed the rats in group G-III with diabetes mellitus type 2, with mild HHcy by biochemical evaluation. The solution was administered only to animals in the G-III after confirmation of the experimental model of diabetes. The control group G-I and the obese G-II received distillate water the same way atorvastatin was given to group III, to account for the human manipulation stress.

Before and after the administration of treatment, we evaluated the biochemical levels of lipid profile, glycemia, OS, and Hcy levels in all three groups. Time point T_0_ is day 24 from start of the experiment. Blood sampling at this point was performed by puncturing with a capillary tube the retro-orbital plexus.

Gross Necropsy: On the 21st day after protocol treatment, the rats had a postmortem examination, which included a rigorous evaluation of the bodies and internal cavities. Euthanasia was conducted on all subjects, followed by cardiac puncture and sampling of 4 mL of terminal blood. The blood was then accumulated in special vacutainer tubes with a clot activator solution for biochemical assays.

### 2.4. Induction of T2DM in an Experimental Model of Rats by Use of Treatment with Streptozocin

T2DM was induced in our experiment using streptozotocin (STZ). In this experimental study, we used the dosage of 35 mg/body weight, and these dosages were adapted to age and weight. The STZ used in the experiment was purchased from Sigma-Aldrich, Merck, Germany. In group G-III, after overnight fasting, all animals’ blood glucose concentrations were measured from the tail vein using a glucometer. Following blood collection, we injected i.p. with only one dose of 35 mg of body weight STZ, solubilized in 0.1 M sodium citrate buffer (at pH 4.5), and administered within 5–10 min of dissolution. The control (G-I) and obese groups (G-II) received citrate buffer solution, i.p.

After that, the G-III group was provided with free access to 10% glucose solution in water to prevent critical hypoglycemia (the most dangerous complication manifesting in the first 24 h after the injection), followed by another measurement of glycemia from the tail vein using a Code-Free glucometer. The model was verified 7 and 10 days later. Rats with blood glucose concentrations of 200 mg/dl or high glucose values were confirmed as diabetic and were included in the experiments. All animals were monitored every day, and weight (g) was evaluated every 7 days. They also had free unlimited access to standard/HFD food and water.

### 2.5. Evaluation of Fasting Blood Glucose Serum Concentration

The levels of blood glucose after fasting in the G-III group were measured using a Code-Free glucometer before and after STZ at 7- and 10-day intervals. Each animal was held in a restrainer, and its tail was massaged to bring sufficient blood to the tip. Then, the end of the tail was meticulously cleaned with an alcohol solution before being punctured with a sterile needle. The drop of blood was collected using a strip and inserted into a glucometer (STANDARD CodeFree Plus^R^, by SD BIOSENSOR^R^).

### 2.6. Measurement of Biochemical Parameters

Approximately 30 min after collection, the blood samples in the vacutainer tubes were centrifuged at 1500× *g* for 15 min at 4 °C. The serum was analyzed to measure a variety of biochemical parameters: glucose, lipid profile—low-density lipoprotein cholesterol (LDL-C), high-density lipoprotein cholesterol (HDL-C), triglycerides (TG), total cholesterol (TC), homocysteine, vitamins B6 and B12 and folic acid. All reagents for the determination of the level of biochemistry parameters and plasma Hcy concentration were procured from BioSystems S.A. (Barcelona, Spain) and Merck. We utilized the automated analyzer ACCENT-200 (PZ Cormay, Warsaw, Poland) based on the manufacturer’s recommendations to validate the results for biochemistry. The antioxidant activity of intracellular and extracellular systems, SOD, GPX, and TAS, was measured using the biochemical analyzer AMP PICCOS 2. The kits for SOD and TAS were procured from Elabscience, while GPx was measured using Randox kits procedures. The total level of plasma Hcy was evaluated by an HPLC-validated method [43]. The chromatographic system consisted of Agilent 1200 HPLC 6520, Binary Pump, Zorbax SB-C 18 (4.6 mm × 250 mm, 5 µm) and UV–VIS detector (DAD). Elution was performed in gradient mode. The sample volume was 20 µL, flow rate 1.2 mL/min, wavelength set at 355 nm, and column temperature maintained at 25 °C.

### 2.7. Statistical Analysis

The data for each biochemical marker were visualized using box plots to represent the interquartile range (IQR) alongside median values. The biochemical parameters were plotted using Python version 3.11 according to the experimental groups (group I, group II, and group III) across two time points (T_0_ = day 24; and day 45). The box plots were generated using the Seaborn library, with the boxes indicating the 25th to 75th percentiles, the line within the box representing the median, and whiskers extending to 1.5 times the IQR. Outliers were visualized as individual points.

The datasets were first assessed for distribution normality using the generated boxplots. For assessing effect of intervention (distribution overlap), we used paired (when comparing between the two timepoints) and independent one-tailed t-test (when comparing two groups at a certain moment) when distributions were close to normal, and Mann–Whitney when curves were far from normal. All figures show the group-respective values, from G-I to G-III, from left to right, at each timestamp, except otherwise stated.

## 3. Results

Regarding weight gain, on a whole-experiment duration (day 0–day 45) the group G-II that were only fed the HFD with cholesterol 2% exhibited a higher growth rate on body mass compared to both the control group (who received only a standard diet) and the diabetic group G-III (G-II, 22.7 ± 4.4 g; G-I, 16.1 ± 3.2 G, *p* = 0.0081; G-III, 17.5 ± 1 g, *p* = 0.01). The statistical difference in weight gain between groups G-II and G-III is not significant when analyzed during the first time slot before the STZ injection (day 0–day 14), the rates being drastically similar (G-II, 23.3 ± 6 g; G-III, 22 ± 4.1 g, *p* = 0.63 >0.05).

### 3.1. Serum Lipid Profile

**Figure 2:**
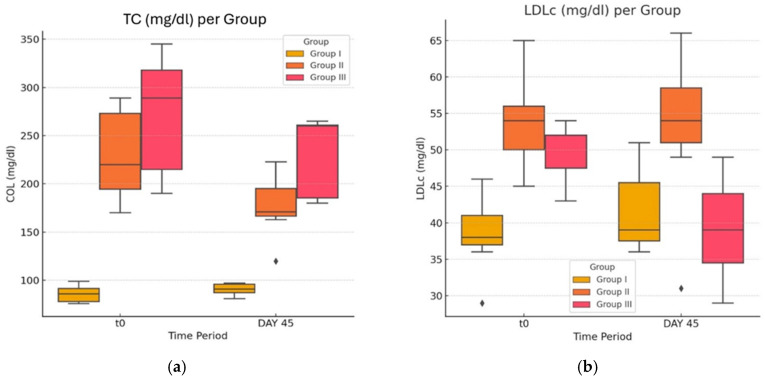
(**a**,**b**) Lipidic profile in normal, obese, and atorvastatin-treated diabetic and obese rats. The ♦ symbol denotes an outlier.

**Figure 3:**
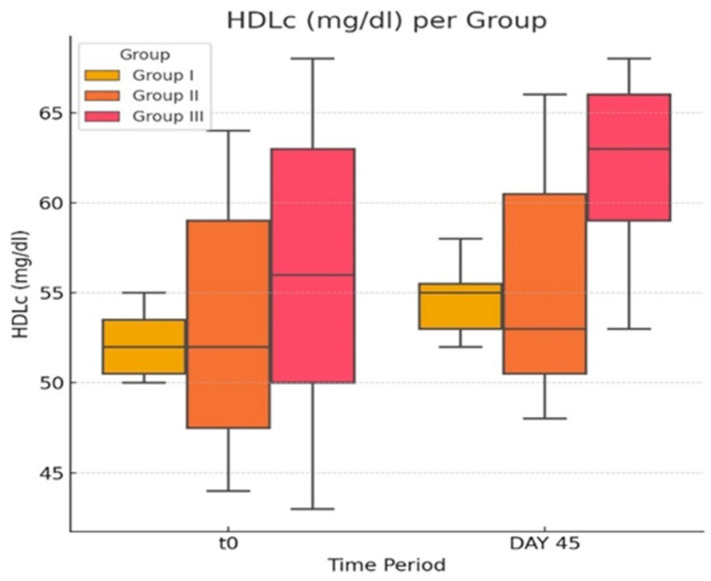
Effect of atorvastatin on HDLc compared across the other groups.

As shown in Figure 2a,b and Figure 3, the obese G-II group has significantly higher concentrations of TG, TC, LDLC, and a lower level of HDLC compared to the normal (control) group. On the whole, the group exposed only to HFD displays a totally worse lipid profile at day 45 compared to the control G-I at the same timestamp.

Focusing on group G-III (benefiting from atorvastatin treatment), these rodents displayed an improvement in the lipid profile, comparing the values of LDLc and HDLc between the two time points T_0_ and day 45. In the case of lipid profile (TC and TG), atorvastatin treatment produces statistically significant (*p* ≤ 0.05) decreases in LDLc values. In the case of the G-III group, we observed increased values of HDLc before and after treatment (G-III—day 45 vs. G-III—T_0_), statistically significant (*p* < 0.05). In contrast, the obese G-II rats did not show any improvement in lipid profile during the same time window. Noticeably, a direct comparison between the LDLc trends in G-II and G-III was significant even after adjusting for the homocysteine serum concentration.

Within group G-I, no statistical difference was found in TC concentrations when comparing the initial to the final experiments. Other variations in values can also be mentioned, but the lack of statistical significance yields no clinical implication (*p* > 0.05).

### 3.2. Effect of Atorvastatin on Triglyceride and Blood Glucose Concentration in Normal, Obese and Diabetic Rats

**Figure 4:**
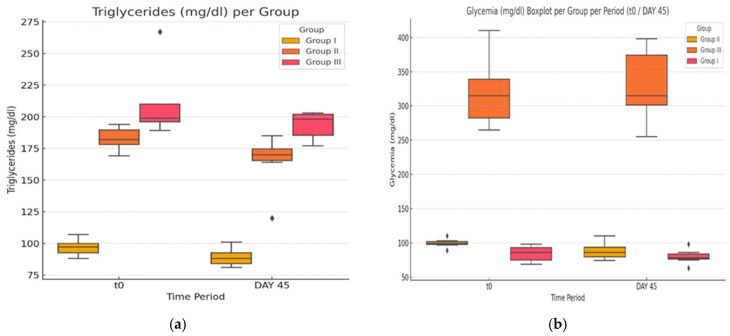
(**a**,**b**) Triglyceride and blood glucose concentration in normal, obese, and atorvastatin-treated diabetic and obese rats. Please note that the middle (orange) group only in (**b**) is the statin-treated G-III group. The ♦ symbol denotes an outlier.

In our groups, in the case of TG levels (Figure 4a,b), we observed that there were statistically significant differences (*p* ≤ 0.05) when comparing the three groups (G-I to G-III) at each of the two time points.

In our model, treatment with atorvastatin did not cause an important change in TG levels in the G-III group, comparing the in-group values before and after the treatment. Slight increases were noted in blood glucose levels, statistically insignificant (*p* > 0.05), in the G-III group, similarly comparing the values before and after the treatment.

### 3.3. Effects of Atorvastatin on Pro-Oxidant Markers in Normal, Obese and Diabetic Rats

**Figure 5:**
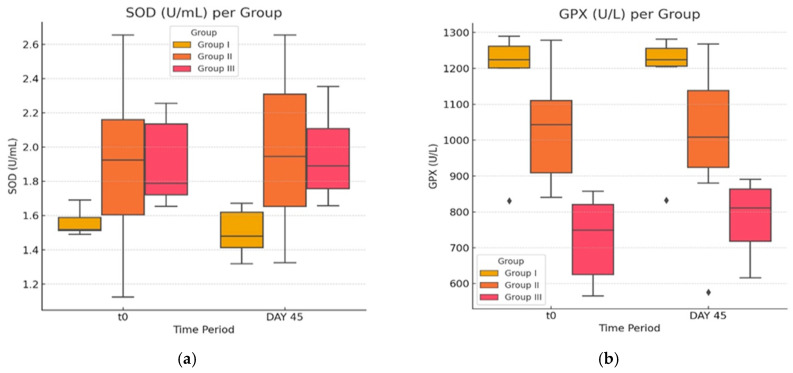
(**a**,**b**) The effect of atorvastatin on glutathione peroxidase activity and superoxide dismutase by assessing the enzymatic capacity initially and after the experiment. The ♦ symbol denotes an outlier.

**Figure 6:**
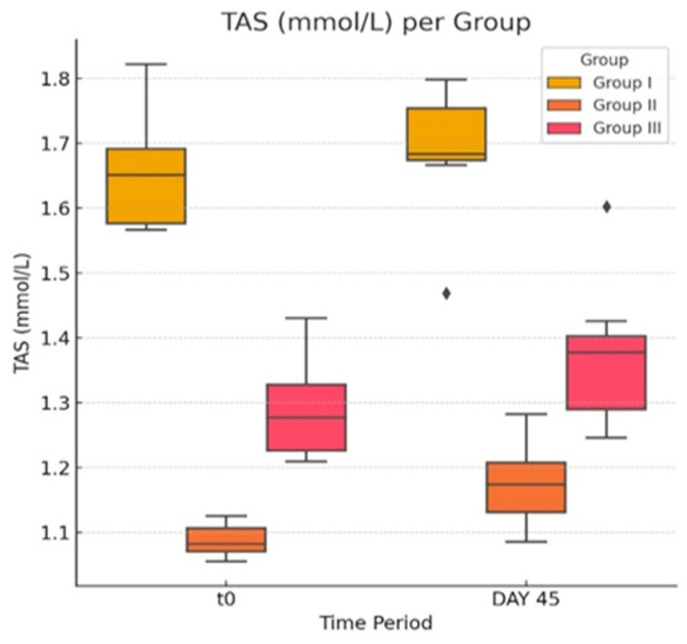
The effect of atorvastatin on total antioxidant status. The ♦ symbol denotes an outlier.

In the second part of the present experiment, we evaluated the effects of atorvastatin on pro-oxidant indicators (Figure 5a,b and Figure 6) in diabetic rats and levels of Hcy. It is generally accepted that HHcy can disturb the antioxidant balance, generate reactive species, and decrease the total antioxidant capacity (TAS).

Comparing the activity of SOD in the G-III group after statin therapy with the initial values, there is no statistically significant change in the value of means (two-tailed paired t-test *p* = 0.73 > 0.05). Neither of the other two groups displays a slight difference in SOD activity when comparing the two time points, yielding no clinical or statistical significance. The present experiment showed statistically significant modifications of parameters in the activity of GPX, in the case of the group treated with statin, between the two moments in time (T_0_, 723.13 ± 118.15; day 45, 782.66 ± 107.41; *p* = 0.04, <0.05). The obese and control group yielded no significant change in parameter GPX. Lastly, TAS showed significant change between the two time points both in group G-III (T_0_, 1.289 ± 0.084; day 45, 1.373 ± 0.120; *p* = 0.034, <0.05) and G-II (T_0_, 1.088 ± 0.027; day 45, 1.174 ± 0.070; *p* = 0.01, <0.05).

In conclusion, while in our study, a few variations in OS parameters can be observed, only the GPX and TAS differences are statistically significant (*p* < 0.05) in the atorvastatin-treated group.

### 3.4. Vitamin B12, B6, Folic Acid and Homocysteine Concentration in Normal, Obese, and Atorvastatin-Treated Diabetic-and-Obese Rats

**Figure 7:**
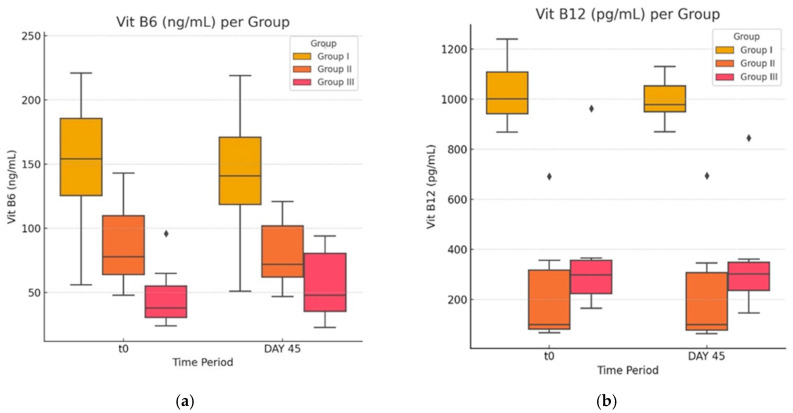
(**a**,**b**) Vitamin levels before and after the experimental study. The ♦ symbol denotes an outlier.

**Figure 8:**
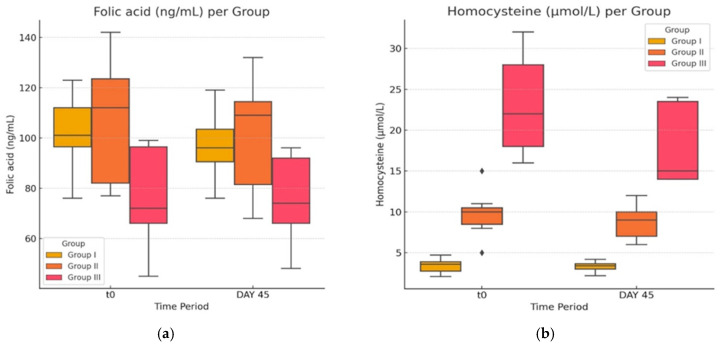
(**a**,**b**) Homocysteine and folic acid levels before and after the experimental study. The ♦ symbol denotes an outlier.

In the last part of our study, we evaluated the impact of atorvastatin on vitamin and Hcy levels. A comparison between the value of means in each of the experimental groups between the initial and final datasets (Figure 7a and Figure 8b) shows that statin treatment had no statistically significant effect on serum levels of vitamin B12, B6, and folic acid (*p* > 0.05).

Only the distribution of the values of vitamin B12 in group G-II was massively skewed to the right in both timepoint datasets. This required the use of the non-parametrical Mann–Whitney test, where the z = 0.127 and *p* = 0.448, way higher than the required value for statistical power.

In spite of the other non-statistical powerful comparisons in these figures between the two time points, in the group treated with atorvastatin, G-III, a statistically significant (*p* = 0.003 < 0.05), albeit minimal absolute reduction in the concentration of Hcy was noted (Figure 8b).

## 4. Discussion

The aim of the current study was to determine the impact of atorvastatin treatment in a rodent obese and diabetic model on the lipidic and oxidative profile, all these revolving around the element homocysteine. As a cardiovascular risk factor, homocysteine has a more defined role, especially in recent years, but surely there is still uncertainty and room for new research, as demonstrated by the recent review in the field [39].

In the current model, there is a systemic burden arising from obesity, and impaired glycemic regulation brings further stress to the cardiovascular system. Atorvastatin, one of the current in-use representatives of the statin class, is amongst the most used drugs for cardiovascular disease. The dosage of atorvastatin was selected in conformity with the current literature; we tailored the dose according to those studies that demonstrated statin’s efficacy in reducing lipemic–oxidative disorders. Recently, it was reported that a dose of 20 mg/body weight/day for at least 20 days was considered efficient and safe (i.e., it has not returned toxicity in rats) [44,45,46,47].

One important point to mention regarding the induction of DM in obese rats has to be made. Most models employing streptozotocin for the induction of diabetes use a single dosage by STZ, administrated i.p., of 60 mg/body weight. However, to account for a presumably already damaged liver by fat deposits in obese rats fed with HFD, our team decided to lower the STZ dose. The rationale was to prevent a high loss of subjects due to the procedure, and the confirmation came on day 24 of the experiment when all animals exposed to STZ revealed a typical DM glycemic profile. The same reduction in STZ dose was performed in several other similar studies [44,48,49,50].

In these experiments, we first evaluated the effect of HFD in every group. Our study showed that administration of HFD—in G-I and G-II—can cause significant weight gain, attracting changes on a biochemical level by increasing TC and TG. In the final stage of the experiment, we observed that group G-III, treated with atorvastatin, exhibited significant lipidic improvement compared to the obese G-II group, and this correlation remained powerful after adjusting for Hcy concentration. The specimens in group G-III showed relatively homogenous Hcy concentrations, all being classified as mild hyperhomocysteinemia. Moreover, the treatment administered over only 20 days in this group proved the hypolipidemic role of statins (G-III). Over the treatment administration period (T_0_ to day 45), when comparing the G-I control group (which received standard food) and obese group (G-II), only the obese and diabetic G-III exhibited decreased levels of TC and LDLc and increased HDLc.

There is no doubt that statins have important benefits in the regulation of TC and LDLc levels. It is now well-established that statins, particularly atorvastatin, considerably influence non-HDL-C and TG values. Statins prevent the storage of cholesterol inside cells by inhibiting the enzyme HMG-CoA reductase. This action stimulates the production of LDL receptors, which increases the rate at which LDL is cleared from the bloodstream, thus reducing plasma cholesterol levels. Recent studies indicate that atorvastatin can decrease TG levels by 10–20% [51,52]. Furthermore, it has been known that the higher the initial TG levels are, the more significant the decrease will turn out; for instance, baseline TG levels exceeding 250 mg/dl have been associated with a decrease between 22 and 45%. However, lower baseline levels see a more modest reduction [52]. While these lipid-lowering effects are observed with various statins, atorvastatin has resulted in more sustainable improvement in plasma LDL and TG compared to others, possibly due to its prolonged action and active metabolites of atorvastatin in the hepatocytes. The TG reduction with statin results from decreased VLDL secretion by the liver and increased elimination of TG lipoproteins through increased LDL receptors in the plasma [53].

Our results agree with previous research, which indicates that 20 mg/kg of body weight/day atorvastatin has lipid-lowering effects. Extensive evidence from clinical studies supports the notion of the “pleiotropic” effects of statins [54,55,56,57,58,59].

In the second part of our study, the main aim was to evaluate the potential results of treatment with atorvastatin on Hcy levels. In our study, the values of Hcy in group G-II could be classified as normal both at baseline and the end of the study if following the currently accepted guidelines in humans. However, in the diabetic group (group G-III treated with atorvastatin), levels of Hcy displayed statistically significant change but were small regarding absolute numbers.

A link between hyperlipidemia and high Hcy levels has been proposed. Elevated plasma Hcy is associated with lower HDLc levels by inhibition of particle formation [60]. Also, the reduced methyl group availability associated with HHcy appears to cause lipid deposits in tissues by lowering the synthesis of phosphatidylcholine, key for VLDL assembly and control. The Hcy values detected in our study could be associated with the role of OS in conveying the actions of Hcy, which is predisposed to form disulfide bonds and produce oxygen-derived free radicals, ultimately promoting the peroxidation of LDL lipids. Statins can improve the prognosis of patients with coronary heart disease and reduce the incidence of major adverse CVD events by improving OS through the upregulation of antioxidative enzymes. Therefore, despite our statistically significant but weak medical approach, the beneficial effect of statin can take place by-passing the homocysteine metabolism. Indeed, various other beneficial effects, such as cardioprotection, improvement of endothelial health, increased nitrous oxide (NO) bioavailability, and antioxidative and anti-inflammatory activities, have been proven in statins, at least in rodent specimens [61].

In another experimental model of diabetic rats, the level of Hcy was associated with dyslipidemia and was decreased by treatment with statin. Atorvastatin improved lipid parameters and decreased hepatic lipid peroxidation, together with an increase in antioxidant markers and a decrease in Hcy levels [36]. Additionally, atorvastatin administration in hyperhomocysteinemic rats decreased the levels of Hcy and lipid profile and increased the serum levels of HDLc in [62]. Treatment with atorvastatin in hypercholesterolemic rats affects the Hcy metabolism, possibly by interfering with glomerular filtration or altering the activity of key enzymes participating in Hcy metabolism, such as cystathionine β-synthase [63].

The central role of homocysteine in the amelioration of potential cardiovascular consequences is revealed by a clinical study on patients with acute coronary syndrome. Despite the overall positive effect of atorvastatin in all patients, higher responsiveness was noticed in those who exhibited the most significant decrease in Hcy levels [64]. Similarly, in a clinical study on 338 subjects, the authors demonstrated that simvastatin treatment caused consistent small reductions in Hcy blood concentrations, with a bigger effect exhibited in the subjects with higher starting Hcy [65]. Whether homocysteine—or, rather, the lack of it—is an enhancer for statins to exhibit their lipid-lowering properties or it is just a surrogate marker for endothelial health is not yet clear.

A study focused on hypercholesterolemic patients reported a decrease in Hcy levels after high-dose simvastatin (80 mg daily) for 24 weeks, suggesting that this precise action could be responsible for the reduction in cardiovascular events, at least in the case of this high-end dosage of simvastatin [66]. The superiority of statin in the management of dyslipidemia patients is unveiled by a triple-arm comparison of two statins (atorvastatin 40 mg/day versus simvastatin 40 mg/day) and fenofibrate upon levels of homocysteine. Their result presented a small change in serum Hcy values only in the statin groups [35]. When compared to groups not receiving treatment, only atorvastatin showed an increase across the whole range of normal to very high concentrations of Hcy; in spite of a potentially beneficial indiscriminate approach, atorvastatin was less effective than simvastatin when the comparison was assessed in patients classified inside a particular stage of HHcy. Therefore, it is likely that different statins might pose different potentials to prevent Hcy-induced OS and apoptosis.

In contrast to these results, some clinical studies reported no changes in Hcy after statin therapy [67,68]. The contradictory findings extend to animal studies, too [69]. This controversy may be due to different statin doses, duration of intervention, different diseases and patient metabolic profiles.

While our experiment finding is in agreement with a small number of studies evaluating the influence of statin treatment on Hcy levels, great care should be taken when interpreting this effect outside the laboratory models [67,68,70,71]. Regarding the effect of statin in humans, a meta-analysis of controlled trials published in 2016 yielded no modification of Hcy concentration following statin therapy [36]. However, an updated meta-analysis from 2024, albeit with a lower significance level due to the inclusion of all types of papers, states a dose- and treatment duration-dependent lowering of Hcy concentration following atorvastatin administration [72].

In the case of glutathione peroxidase (GPX), our results proved statistically significant differences between the three examined groups analyzed at time T_0_, which maintained significance at the end of the study. This relation also occurs inside the group with atorvastatin treatment when comparing the values at the beginning of the study with the ones at the end. The in-group differences between G-I and G-II were not significant.

In our experiment, we observed that TAS was significantly increased in the G-III group at the end of treatment. Disconcerting to our observation is that a statistically significant difference has also been noted in the datasets of group G-II.

SOD are important endogenous antioxidant enzymes of first-line defense that catalyze the dismutation of superoxide radicals; in our experiment, the results showed that there was no difference between time points inside any of the groups regarding the activity of SOD. There is, however, a mathematically significant difference at the starting time point between the control and any of the working groups, a difference which maintains significance by the end of the study. This is not relevant to atorvastatin properties. Concerning our study, compared to the group without treatment, atorvastatin alone could not return an increased concentration of SOD; there was a statistically significant variation, albeit with weak medical relevance in the Hcy values. Our results do not suggest that treatment with atorvastatin can offer protection against OS caused by disorders of lipid metabolism.

TAS is characterized by the capacity to neutralize free radical burden, serving as a measure of the productivity of cellular antioxidant systems. A decrease in TAS concentration indicates an increase in oxygen free radicals, with a decline in the capacity of the antioxidant defense mechanism. Additionally, TAS can reflect the antioxidant potential of drugs and determine if a new treatment has efficacy on the body’s antioxidant system [73].

Potential antioxidant mechanisms of atorvastatin include reducing ROS production by inhibiting vascular NAD(P)H oxidase, altering redox balance in LDL particles, modulating RNA expression, increasing NO synthesis in blood vessels, and attaching to the surface phospholipids of lipoproteins [74,75,76].

Previous studies concerning both in vivo and in vitro experiments raised controversy regarding the actual involvement of statins in determining cardiovascular benefit. For example, it was proven that high-dose atorvastatin can influence the activity of GPx and catalase [77,78]. Similarly, in another experimental study HATA, increased activities of GPx and SOD were noted in rats treated with high-dose atorvastatin [79]. Moreover, another experimental research observed that simvastatin is more efficient than atorvastatin in HHcy rats in ameliorating oxidative stress biomarkers after 4 weeks of treatment [80]. A similar study in obese rats reported that simvastatin ameliorated OS by increasing the levels of antioxidant enzyme activities [37]. Furthermore, concurrent data about the impact of simvastatin and vitamin D supplements on OS demonstrates that treatment with both has a positive impact on OS and inflammation in obese rats [38].

Therefore, treatment with atorvastatin confirms the results of other studies, which show that it has hypolipidemic, antioxidative, and antiatherosclerotic effects. The American Heart Association/American College of Cardiology (AHA/ACC) guidelines recommend high- or moderate-intensity treatment with statin for patients diagnosed with diabetes mellitus or intermediate to high cardiovascular risk [81,82,83]. In medical practice, atorvastatin is used to prevent CVDs. Recent data demonstrate the efficacy, benefits, and tolerability of different formulations of atorvastatin for patients with hypercholesterolemia [82,83]. A clinical study in patients with hypercholesterolemia and high risk for CVD demonstrated the efficiency of 20 mg/day atorvastatin in reducing LDL-C and cost-effectiveness, compared with atorvastatin 10 mg/day [84]. Different formulations of the same substance seem to yield the same efficacy and no difference in the rate of adverse effects [85].

Nutritional or therapeutic treatment approaches have been suggested for the therapy of HHcy, but the results of large clinical research aimed at understanding how reducing Hcy levels and impacts of cardiovascular risk are still disputable.

Our findings support the hypothesis that atorvastatin has cardiovascular protective effects, including modulation of endothelial function, reduction of OS, and anti-inflammatory properties, which is consistent with the literature, but more extensive clinical trials are needed [78,86,87].

Although statins are known to lower lipids and homocysteine (Hcy) levels, it is unclear whether atorvastatin’s lipid-lowering effect and reduction of Hcy levels occur independently or if there is an interaction between them. Maybe further trials are recommended to clarify the Hcy-lowering effect in treatment with atorvastatin to prevent cardiovascular events.

Several limitations should be acknowledged in the present study. Firstly, statin therapy was administered only in the experimental studies of diabetes mellitus type 2, not in rats with obesity, in a small number group, and on a fixed rather short-term. Secondly, we could not examine the effects of other statin molecules, different dosages of statin, or different treatment periods. The mathematical analysis had to be carefully interpreted due to the small sample size and, in some cases, the extreme skewness of the distribution. Despite the limitations of the present experiment, our results could contribute to the prevention therapy of major adverse cardiovascular events, attending to an increased awareness towards Hcy levels in clinical practice.

## 5. Conclusions

In conclusion, our results confirm that therapeutic dosages of atorvastatin can be used not only as a hypolipidemic and antioxidant drug but also to reduce Hcy concentration in CVDs, which is in favor of their cardioprotective properties. Statins could ameliorate atherosclerosis development, possibly by lowering the lipids profile, inhibiting inflammation, suppressing oxidation, decreasing the Hcy levels, and preventing cardiovascular diseases.

Therefore, understanding the mechanisms and the association between HHcy levels and oxidative damage could be a basic start for developing new treatment strategies for the prevention of cardiovascular disease.

Future clinical studies and new treatment strategies should be focused on lowering both Hcy and lipid profile levels, preserving the redox balance, and interfering with risk factors for cardiovascular mortality, interventions which will need a long time and clinical, prospective, or placebo-controlled trials.

## Figures and Tables

**Table 1:** The experimental animal groups and the treatment/dosage received.

Group	Name	Treatment	No. of Animals (*n*)
G-I	Normal control	Standard diet	7
G-II	Obese group	High-fat diet (HFD) with 2% cholesterol	7
G-III	Diabetic mellitus animals	High-fat diet (HFD) with 2% cholesterol + atorvastatin 20 mg/of body weight/day, orally, by gavages for 20 days	7

## Data Availability

The data presented in this study are available on request from the corresponding author.
